# Clinical Reports on Continued Fever

**Published:** 1852-09

**Authors:** 


### EDITORIAL.

*Clinical Reports on Continued Fever.*

PROFESSOR AUSTIN FLINT, of the University of Buffalo, and editor of the Buffalo *Medical Journal*, is the author of a work of 390 pages, on Continued Fever as observed in Buffalo and North Boston, Erie Co., N. Y. The work embraces three clinical reports —the first, based on an analysis of fifty-two cases—the second, forty-eight cases—and the third, sixty-four cases—giving in all the histories of one hundred and sixty-four cases. The cases were divided into three classes, *typhus*, *typhoid*, and *doubtful*—and after a careful analysis of each division separately, the results are compared with each other and general inferences drawn. The work was written in sections, and published from time to time in the Buffalo *Medical; Journal* which fact, we presume, will account for any want of method in arrangement.

A supplement to the reports contains the following:

SUMMARY OF SYMPTOMS DISTINCTIVE OF TYPHOID AND TYPHUS FEVERS.

*Age.* The maximum, and mean age higher in *typhus* than in *typhoid.*

*Nativity.* In *typhoid,* patients foreign immigrants from different countries, with a certain proportion of citizens of this country. In *typhus,* foreign immigrants, except when the disease is communicated by contagion, and almost invariably the immigrants recently from Ireland.*

*Season.* The liability to *typhoid* much greater during the autumnal months; *typhus* equally and even more apt to occur during other portions of the year.

*Duration of disease prior to admission into hospital.* In a marked degree shorter in *typhus* than in *typhoid.*

*Symptoms of access. Diarrhœa* present in a certain proportion of cases in *typhoid* during the access, not in *typhus.*

*General aspect.* Capillary congestion of the face, extending frequently to the extremities, and more or less over the body,

* It will be borne in mind that this summary embraces the conclusions deduced from the facts contained in the previous reports. Were it extended so as to embrace conclusions deduced from facts contained in other analyses, it would not only comprehend additional distinctive symptoms, but some of those which it embraces might require qualification. This note is suggested by what is stated under the head of nativity.

causing a dull red color, present in *all* cases of *typhus*, and in a large proportion of the cases of *typhoid*; but the congestive redness greater in *typhus* than in *typhoid*, and presenting in some cases of the former type a dusky and dingy hue.

NERVOUS SYSTEM. *Passive delirium*, manifested by incoherent talking, muttering, attempting to get out of bed, etc., present more constantly in *typhus*; developed earlier in the progress of the febrile career in *typhus*; but very active, persistent delirium, requiring forcible restraint, characteristic of *typhoid*.

*Cephalalgia*, oftener present after the fever becomes established, and longer in duration in *typhoid*, (? see second report.)

*Vascular injection of conjunctiva* oftener present in *typhus*, (? see first report.)

*Deafness*, present in a proportion of cases larger in *typhus*.

DIGESTIVE SYSTEM. *Appetite*, or relish for food, oftener present in *typhus*, (see second report.)

*A reddened tongue*, occasionally observed in *typhoid*, and not in *typhus*.

*Sordes*, present in a large proportion of cases of *typhus*.

*Vomiting*, more apt to occur in *typhoid*.

*Diarrhœa*, present in *one-half* of the cases of *typhoid*, and in *one-third* of the cases of *typhus*; in the latter type always mild or slight, but in the former sometimes prominent as a symptom.

*Hæmorrhage from the bowels*, characteristic of *typhoid*.

*Tympanites*, present in about an equal ratio in both types; but in *typhus* almost invariably slight, while in *typhoid* it is often prominent.

*Tenderness on pressure over the abdomen*, an almost constant symptom in *typhoid*, and less frequently present in *typhus*. In the latter usually slight, and in the former more apt to be marked, or considerable in degree.

*Peritonitis from perforation of intestine*, peculiar to *typhoid*.

*Eruption*. An eruption present in a larger proportion of cases of *typhus*; more abundant in *typhus*, frequently very copious, and extending over the upper extremities, as well as on the trunk; in *typhoid* seldom copious, often only sparely scattered over abdomen and chest, and not present in the extremities. The characters of the eruption as follows: In *typhoid*, rose-colored, oval, slightly elevated, redness momentarily disappearing on pressure. In *typhus*, of a dull red color, smaller in size, not elevated, redness imperfectly disappearing on pressure. These characters preserved in the two types in the great majority of cases. Occasionally slight variations, and some intermingling of the two kinds of eruption.

RESPIRATORY SYSTEM. *Cough* more uniformly present in *typhus* (?)

*Pneumonitis* more apt to be developed in *typhus* (?)

*Epistaxis* extremely rare in *typhus*, occurring frequently in

*typhoid.* Sputa detached from the posterior nares apt to be tinged with blood in *typhoid*, not in *typhus*.

CIRCULATION. Greater frequency of the pulse in *typhus*, than in *typhoid*. The average frequency in the former exceeding an hundred per minute, in the latter falling below that number. Instances of great increase in frequency occurring oftener in *typhus*.

SKIN, exclusive of eruptions and congestive redness, not presenting symptoms distinctive of either type.

*Duration*, longer, before death, or convalescence, in *typhoid*, than in *typhus*, this rule being subject to variations, owing to an unusually short duration of the *typhoid* type at some periods. (See second report.)"

The results of these analyses, while they show a great similarity between the forms of *typhus* and *typhoid*, seem to favor the doctrine of their non-identity.

*Treatment.* Full doses of opium were administered in several cases with the apparent effect of shortening the course of the disease. Quinine seemed to have no influence whatever. The wet sheet, or "packing," was used in some instances with improvement of symptoms. In cases accompanied with delirium, the author has obtained the best results from the use in small doses of the tartrate of antimony and potassa in connection with anodynes. In regard to the use of cathartics, he has the following judicious remarks:

The question here suggests itself, what are the indications in the management of the disease which pertain to cathartics? Most writers on this subject enjoin the employment of remedies of this class sufficiently to procure one or more dejections daily; and the majority of practitioners, probably, make this an object of treatment. My own observations lead me to hold a different view. The results of the investigations of the histories that I have collected show, *first*, that diarrhœa frequently follows the operation of a cathartic; *second*, that the proportion of cases characterized by diarrhœa is greatest among those in which cathartics were employed; *third*, in one case hæmorrhage from the bowels followed the operation of doses of castor oil, occurring only in this connection, and no cathartics being given in that case with these exceptions; and, *fourth*, no appreciable evils resulted from omitting all remedies of this class in a large proportion of the cases, a dejection not occurring, in some instances, for periods varying from three to eight days.

These facts appear, to my mind, to render more than doubtful the propriety of resorting to cathartics in continued fever, save for some special objects.

Looking at the subject rationally, it is to be considered, *first*, that cathartics are not indicated, more than other remedies, by the mere fact of fever being present. We have no adequate evidence that patients pass through the disease more safely and pleasantly, other things being equal, when cathartics enter into the management, than when they do not. *Second*, it is a principle of general application in therapeutics, not to be lost sight of here, more than in other affections, that remedies of more or less potency, if not indicated, will be likely to prove hurtful. If not efficient for good, they will probably be productive of harm in proportion to their activity. They cannot be expected to be neutral in their consequences. *Third*, it is not difficult to conceive, from the *modus operandi* of cathartic remedies, how they may prove injurious. Their local action on the alimentary canal involves more or less irritation, and this, when the follicular patches are diseased, ulcerated, in the *typhoid* type, (a condition not incompatible with constipation,) cannot but be prejudicial. Under these circumstances the only way in which they can be considered appropriate in their local effects is to suppose that by effecting the discharge of the morbid contents of the intestinal canal, they remove causes of irritation, which, by remaining, would do greater harm than is occasioned by the action of the cathartic. Admitting that the direct effects of the latter are bad, it may be said that it is indirectly useful by causing the discharge of what would occasion effects much worse. This is doubtless the reasoning often adopted; but the premises are assumed, not proved. Indeed, in so far as the facts presented in the preceding reports furnish evidence relating to the matter, it is adverse to the hypothesis just stated. Other reasons which may be assigned, such as 'to correct the secretions,' 'to excite the action of the liver,' etc., are too gratuitously speculative to deserve attention, more particularly so long as the results of experience appear to be adverse to the utility of cathartics.

The remote, or general effects of this class of remedies is to diminish the vital forces, to conduce to prostration. In other words they are debilitating in their tendency, and, hence, it is reasonable to suppose that they may be productive of harm rather than good. If the bowels do not move spontaneously after several days, even if no evils are apparent, it may be the part of prudence to effect a movement. But for this, so far as my experience goes, active cathartics are not requisite. Remedies distinguished as *laxatives* will suffice, and even these, simple injections will usually render unnecessary. The latter are always to be preferred when practicable or adequate.

The author thinks bleeding rarely indicated, and less frequently in the *typhus* than in the *typhoid* type. Stimulants were used freely in the cases reported, and are recommended—a half an ounce of brandy with an equal quantity of water, repeated as often as may be necessary to sustain the powers of the system—this was almost the only medication in the third collection of cases. An important item in the treatment, by Dr. Flint, is the systematical administration of food without reference to the appetite or wishes of the patient.

This disease is characterized by debility, and whatever difference of opinion may exist regarding the propriety of alcoholic stimulants, we think there can be none on the question of supplying a nutritious diet. The powers of the system need sustaining, the muscular tissues need nourishing, and the essence of beef which the author gives in connection with milk porridge, is the substance most nearly allied to the suffering structures, as well as most easily assimilated. If to these articles *common salt* be *freely* added, we can conceive of nothing more perfectly adapted to meet the indications in typhus and typhoid fever. These diseases are increasing in frequency in the West; cases of typhoid, especially, are common in the wards of our hospitals, and it seems not improbable that our periodical fevers will, at no distant day, give place to those of a purely continued type; the book of Dr. Flint is, therefore, a timely one.

Our limits do not permit us to accompany the author further, but we cannot close without referring the reader to the chapter on the *transportation* and *diffusion* of typhoid fever. Facts are detailed which go to show that, under some circumstances at least, it may become highly *infectious* if not *contagious*.

The publishers are Geo. H. Derby & Co., Buffalo; Geo. P. Putnam, 10 Park Place, New York; D. B. Cook & Co., (successors to Hewson & Denison,) 135 Lake-st., Chicago.

We have read the work with pleasure, and commend both author and publishers to the favorable consideration of our readers. J.

